# Depressive symptoms among Chinese residents: how are the natural, built, and social environments correlated?

**DOI:** 10.1186/s12889-019-7171-9

**Published:** 2019-07-05

**Authors:** Ruoyu Wang, Ye Liu, Desheng Xue, Marco Helbich

**Affiliations:** 1 School of Geography and Planning, Sun Yat-Sen University, Xingang Xi Road, Guangzhou, 510275 China; 2 Guangdong Key Laboratory for Urbanization and Geo-simulation, Sun Yat-Sen University, Xingang Xi Road, Guangzhou, 510275 China; 3 Department of Human Geography and Spatial Planning, Utrecht University, Utrecht, The Netherlands

**Keywords:** Depression, Built, natural, and social environments, Moderating effect, China

## Abstract

**Background:**

Depression has become a severe societal problem in China. Although many studies have analyzed how environmental characteristics within neighborhoods affect depression, only a few have dealt with developing countries, and even fewer have considered built, natural, and social environments concurrently.

**Methods:**

Based on a sample of 20,533 Chinese residents assessed in 2016, the present study examined associations between depressive symptoms and respondents’ built, natural, and social environments. Depressive symptoms were measured using the Center for Epidemiologic Studies Depression Scale (CES-D), and multilevel regression models were fitted accounting for potential covariates.

**Results:**

Results indicated that living in neighborhoods with more green spaces and a higher population density were negatively associated with CES-D scores. Living in neighborhoods with more social capital was protective against depression. Furthermore, results showed that the social environment moderated the association between the built environment and depression.

**Conclusions:**

Social environments moderate the relationship between the built environment and depression. As environments seem to interact with each other, we advise against relying on a single environment when examining associations with depressive symptoms.

## Background

The prevalence of depressive symptoms is a serious societal challenge in China. The most recent China Health and Retirement Longitudinal Study [1] showed that depressive disorders are widespread across the population (lifetime prevalence is approximately 25%).

Significant efforts have been made to unravel risk and protective factors for depression. In the past, for instance, the emphasis was primarily on individual determinants, such as gender, age, psychological conditions, etc., but research interest has shifted towards the contribution of environmental characteristics [2, 3]. There are diverse environments, including natural environments (e.g., green spaces such as parks and forests; blue spaces such as lakes and rivers) [4–7], environmental hazards (e.g., noise and air pollution) [8, 9], built environments (e.g., urban form) [10, 11], and social environments (which encompasses social relationships and cultural milieus within which people interact) [12] that could influence depressive symptoms. When investigating risk and protective factors for depression, it is likely that environmental factors are at play [13]. Although environmental exposures occur elsewhere (e.g., in the workplace) [14], immediate residential context is central to people’s daily activities, thereby playing an important role in affecting depression risk [15–24].

Findings addressing the contribution of environmental factors on depression are not consistent across studies. This is at least partly due to the following reasons. First, previous studies on the link between environmental factors and depression mainly focus on one specific environmental category (i.e., the natural environment, environmental hazards, the built environment, or the social environment) instead of multiple categories. For example, a recent cross-sectional, ecological study explored the impact of green spaces on depression, without taking the social environment into account [25]. This may lead to omitted variable bias, namely that relevant variables are missing from the model while having a significant influence on the mental health outcome.

Second, the majority of previous studies are limited to the direct health effect of environmental factors and disregard the fact that different categories of environmental factors may interact with each other. It is plausible that different environmental factors enhance health benefits or alleviate negative health threats [24]. For instance, it remains largely unclear whether the social environment moderates the relationship between the natural and the environment on mental health [26, 27]. Some studies have noted that the social characteristics of a neighborhood can moderate the relationship between residents’ built and/or natural environment on depressive symptoms [13, 28]. Wang et al., for example, found that neighborhood social capital played a role in weakening the effect of air pollution on depressive symptom risk [13]. Nevertheless, whether a neighborhood’s social environment also moderates the relationship between residents’ depressive symptoms and the built and natural environment is still unknown and needs further investigation.

The present study addressed these significant knowledge gaps by investigating the extent to which the built environment, the natural environment, and the social environment are associated with depression risk in China. We paid special attention to interdependencies between the social environment, the natural environment, and the built environments in shaping residents’ depressive symptoms. The structure of this paper is as follows. The following section provides some background information regarding the study sample, data, and methods. Our statistical results are then presented and embedded in current debates. Finally, the paper concludes with a brief summary of key findings.

## Methods

### Study sample

This study utilized a cross-sectional design based on nationally representative data from China. We used survey data from the 2016 wave of the China Labor-force Dynamics Survey (CLDS). In this survey, respondents were sampled using a multistage, cluster, stratified, probability proportional to size sampling technique. The sampling procedure was conducted as follows. First, the survey team chose 158 prefecture-level divisions in 29 provinces in a random fashion. Prefecture-level divisions are the secondary administrative divisions and include prefectures, prefecture-level cities, and leagues. Second, 394 neighborhoods were randomly chosen from the prefecture-level divisions. Third, 14,226 households were randomly chosen from the neighborhood-level divisions. Household members who were aged between 15 and 64, and who were aged above 64 but were involved in the labor force, entered the sample. The response rate was 86.15% (91.35% for rural areas and 80.20% for urban areas). After omitting cases with missing data and invalid questionnaires (*n* = 328), our final sample included 20,533 people living in 394 neighborhoods.

### Data

#### Dependent variable

The outcome variable was experienced symptoms associated with depression, assessed with the Center for Epidemiologic Studies Depression Scale (CES-D). The CLDS questionnaire contains a CES-D scale, and all respondents were invited to complete this measure. The CES-D comprises 20 items and is designed to measure the mental state of residents over the previous week (i.e., feeling alone, feeling disliked, and people being unfriendly) [29]. The CES-D score ranges from 0 to 60, with higher values indicating higher depressive symptoms. The CES-D has been shown to have good validity and reliability within Chinese samples [30].

The Cronbach’s alpha of 0.95 on the CES-D suggested excellent internal consistency of the measure within the current sample. Concurrent validity was evaluated by examining factor loadings from a confirmatory factor analysis. All factor (item) loadings were greater than 0.50 and significant at the 0.05 level, indicating that the CES-D had adequate concurrent validity. The average variance extracted from each item was greater than the squared correlation coefficients between items, suggesting adequate discriminant validity. To produce a more normally distributed CES-D score, we applied a log transformation before the variable was included in the regression analyses.

#### The natural outdoor environment

The CLDS also contains questions about both individual attributes and the environmental characteristics of the community. As for the natural outdoor environment, we considered residents’ exposure to green spaces in the analyses, which was measured by the percentage of greenery coverage within the neighborhood. Specifically, trained auditors were hired by the CLDS survey team to measure residents’ exposure to green spaces based on the digital map of each neighborhood [25]. We assumed that people who were more exposed to green spaces would have fewer depression symptoms [31].

#### The built environment

The built environment was operationalized in two ways. As the prevalence of psychiatric disorders tends to vary across urban and rural areas [32], we considered urbanicity in our analyses. Urbanicity was operationalized as the population density measured by the number of people per unit area. We assumed a positive correlation between neighborhood population density and urbanicity. The presence of sports facilities within a neighborhood has been noted to lower the risk of mental disorders through the encouragement of physical activities [33]. Thus, we used a dummy variable to capture the existence of sports facilities within the neighborhood.

#### The social environment

Five variables were used to capture quality of the neighborhood social environment, which was aggregated from individuals to the neighborhood level. We used social trust, social reciprocity, and social group membership to measure neighborhood social capital [34, 35]. Social trust is part of the CLDS survey and was measured as the proportion of residents within a neighborhood who reported that people living in the neighborhood were trustworthy. Neighborhood-based social reciprocity was quantified by the proportion of residents within a neighborhood who found their neighbors supportive and were willing to help each other. Social group membership was measured by the proportion of residents within a neighborhood who belonged to at least one of the following social groups: *ju wei hui* (residents’ committee), social work organization, homeowners’ association, leisure/sports group, *tong xiang hui* (townsman association), clan organization, volunteer organization, and religious groups. To quantify the level of inequality in the average annual household income per household member, we calculated the Gini index [26, 36]. An index of 0 refers to equal distribution of income, while 1 refers to maximum income inequality. Finally, we considered neighborhood security, assuming that respondents living in insecure areas were more likely to experience depressive symptoms [26]. This variable was operationalized as the proportion of residents within a neighborhood who reported that their neighborhood was safe.

#### Covariates

Following previous studies [2, 3], we adjusted for a series of individual-level and neighborhood-level covariates. These covariates were gender, age, marital status, educational attainment, employment status, hukou status, living area, smoking history, drinking history, medical insurance status, physical health condition, neighbourhood type (urban and rural), individual-level social capital (trust, reciprocity, and social group membership), individual sense of security, annual household income per capita, and annual neighborhood income per neighborhood resident.

### Statistical analyses

Summary statistics were used to describe the data. As regression assumptions are challenged by multicollinearity, we used variance inflation factors (VIF) to test for this possibility. VIF scores larger than three indicate suspicious correlations among covariates. Based on the VIF scores in this study (< 3), we found no evidence that multicollinearity among variables was an issue. Subsequently, we assessed the associations between CES-D scores and the environmental variables. Due to the hierarchical structure of data, whereby people are nested in neighborhoods, it was necessary to apply multilevel modeling [37] to avoid biasing the model output. The intra-class correlation coefficient of the null model (i.e., 0.17) showed that CES-D scores within the same neighborhood were somewhat correlated, confirming the application of multilevel models.

A set of multilevel models with different levels of complexity were fitted. Our null model (i.e., one without covariates) quantified the degree of intra-class correlations across neighborhoods. Our baseline model (Model 1) assessed the correlations between CES-D scores and the environmental variables while adjusting for the covariates. Next, cross-level interactions were added to Model 1 to identify whether the social environment moderated the relationship between the built and the natural environment, as well as respondents’ depression level (Models 2–4). These models were fitted with a random intercept per neighborhood. To compare the quality of these models, we employed the Akaike information criterion (AIC). Lower AIC scores indicate better model fit. All continuous independent variables were mean centered.

## Results

Summary statistics for all variables are presented in Table 1. The mean CES-D score for all respondents was 7.28, with a standard deviation of 9.26.

**Table 1:** Summary statistics of the variables

	Proportion	Mean (SD)
Variable		
CES-D score		7.28 (9.26)
Natural environment of the neighborhood		
Green space (%)		49.73 (27.29)
Built environment of the neighborhood		
Urbanicity (people/km^2^)		29,625.05 (415,537.75)
Sports facilities (yes, no)	66, 34%	
Social environment of the neighborhood		
Social trust		0.78 (0.12)
Social reciprocity		0.48 (0.23)
Social group membership		0.08 (0.15)
Gini index of household income		0.43 (0.10)
Security		0.91 (0.10)
Covariates at the individual level		
Gender (male, female)	47, 53%	
Age (years)		44.81 (14.61)
Marital status		
Single, divorced, or widowed	19%	
Married – living with spouse	73%	
Married – not living with spouse	8%	
Educational attainment level		
Primary school or lower	35%	
High school	52%	
College or higher	13%	
Employment (employed, unemployed)	95, 5%	
*Hukou* status (local *hukou,* non-local *hukou)*	91, 9%	
Smoker (yes, no)	27, 73%	
Alcohol use (yes, no)	19, 81%	
Medical insurance (yes, no)	90, 10%	
Physical health status (good, bad)	90, 10%	
Neighbourhood type (urban neighbourhood, rural neighbourhood)	38, 62%	
Social trust (trustworthy, not trustworthy)	22, 78%	
Social reciprocity (people often help each other, seldom/never)	48, 52%	
Social group membership (number of social groups)		0.08 (0.37)
Sense of safety (safe, unsafe)	91, 9%	
Average annual household income per household member (CNY)		18,010.46 (204,076.28)
Average annual neighborhood income per neighborhood resident (CNY)		17,826.38 (32,388.29)

Table 2 shows the estimation results for all regression models. Starting with Model 1, which considered the main effects only, CES-D scores were significantly related with the quality of the natural, the built, and the social environment. Specifically, green spaces [Coef. = − 0.045, SE = 0.023] and urbanicity (i.e., logged population density) [Coef. = − 0.042, SE = 0.015] were inversely related to CES-D scores. Social trust [Coef. = − 0.407, SE = 0.205], reciprocity [Coef. = − 0.286, S E = 0.120], and group membership [Coef. = − 0.404, SE = 0.159] were consistently negatively related to CES-D scores. These correlations were statistically significant. No evidence showed that availablity of sports facilities, security or Gini index was associated with CES-D scores. The majority of covariates revealed the expected results, and their relationships with depression were, with a few exceptions, statistically significant. No evidence was found for either smoking or medical insurance being associated with CES-D scores at the 5% significance level.

**Table 2:** Results of the multilevel regressions

DV: Logged CES-D score	Model 1	
	Coefficients	Standard error
Natural environment of the neighborhood		
Logged green space	−0.045*	0.023
Built environment of the neighborhood		
Urbanicity	− 0.042**	0.015
Sports facilities (ref: no)	−0.001	0.044
Social environment of the neighborhood		
Social trust	−0.407*	0.205
Social reciprocity	−0.286*	0.120
Social group membership	−0.404*	0.159
Gini index of household income	0.209	0.195
Security	0.325	0.217
Covariates		
Male (ref: female)	−0.190**	0.019
Age	0.003**	0.001
Marital status (ref: single, divorced, widowed)		
Married – living with spouse	−0.116**	0.022
Married – not living with spouse	−0.100**	0.033
Educational level (ref: primary school or lower)		
High school	−0.134**	0.019
College or higher	− 0.098**	0.031
Employed (ref: unemployed)	−0.101**	0.033
Local *hukou* (ref: non-local *hukou*)	−0.063*	0.031
Smoker (ref: no)	0.015	0.022
Alcohol use (ref: no)	0.047*	0.021
Medical insurance (ref: no)	−0.036	0.025
Physical health status (ref: good)	0.674**	0.025
Urban neighbourhood (ref: rural neighbourhood)	0.116*	0.058
Logged household income per capita	−0.061**	0.008
Social trust (ref: not trustworthy)	−0.212**	0.019
Social reciprocity (ref: people seldom/never help)	−0.120**	0.017
Number of social groups	−0.384**	0.033
Sense of security (ref: unsafe)	−0.200**	0.027
Logged community income per capita	−0.080**	0.058
Constant	3.971**	0.345
Var. (constant)	0.128**	
Var. (residual)	1.074**	
AIC	60,554.29	

Significance level: * *p* < 0.05, ** *p* < 0.01. All continuous independent variables were mean centered

Models 2–4 extended the baseline model by adding cross-level interaction terms between the significant neighborhood-level environmental variables. In general, the results indicated that the social environment can either strengthen or weaken the natural environment-depression relationship, as well as the built environment-depression relationship. Model 2 showed that neighborhood-based social capital strengthened the relationships between green spaces and CES-D scores. The income-based Gini index weakened the association between green spaces and CES-D scores. Model 3 showed that both social capital and a sense of security strengthened the relationship between urbanicity and CES-D scores. In contrast, the Gini index of income weakened the relationship between urbanicity and CES-D scores. Similarly, Model 4 indicated that social capital strengthened the relationship between the presence of sports facilities and CES-D scores, while the income-based Gini index weakened the association between the presence of sports facilities and CES-D scores (Table 3).

**Table 3:** Results of the multilevel regressions with interaction effects

DV: Logged CES-D score	Model 2		Model 3		Model 4	
	Coefficient	Standard error	Coefficient	Standard error	Coefficient	Standard error
Natural environment of the neighborhood						
Green space	−0.045*	0.023	− 0.039*	0.019	− 0.047*	0.023
Built environment of the neighborhood						
Urbanicity	−0.046**	0.015	−0.031*	0.015	−0.048**	0.015
Sports facilities (ref: no)	−0.008	0.044	−0.020	0.043	−0.024	0.047
Social environment of the neighborhood						
Social trust	−0.400*	0.206	−0.402*	0.203	−0.838*	0.358
Social reciprocity	−0.375**	0.121	−0.396**	0.118	−0.379*	0.129
Social group membership	−0.405*	0.160	−0.363*	0.158	−0.501**	0.165
Gini index of household income	0.289	0.198	0.107	0.195	–0.910**	0.213
Security	0.381	0.219	0.0.337	0.218	−0101	0.477
Neighborhood-based interaction effects						
Green space × social trust	−0.343*	0.135				
Green space × social reciprocity	−0.413**	0.128				
Green space × social group membership	−0.395*	0.164				
Green space × Gini index of household income	0.665**	0.101				
Green space × security	−0.346	0.320				
Urbanicity × social trust			−0.128**	0.041		
Urbanicity × social reciprocity			0.089	0.058		
Urbanicity × social group membership			−0.277**	0.054		
Urbanicity × Gini index of household income			0.218**	0.071		
Urbanicity × security			−0.249*	0.097		
Sports facilities (ref: no) × social trust					0.267	0.440
Sports facilities (ref: no) × social reciprocity					−0.455**	0.078
Sports facilities (ref: no) × social group membership					−0.426*	0.210
Sports facilities (ref: no) × Gini index of household income					2.281*	0.151
Sports facilities (ref: no) × security					0.481	0.527
Constant	3.912**	0.346	3.876**	0.341	5.100**	0.538
Var (constant)	0.129**		0.123**		0.135**	
Var (residual)	1.071**		1.022**		1.059**	
AIC	60,505.86		59,552.09		60,295.20	

Significance level: * *p* < 0.05, ** *p* < 0.01. All continuous independent variables were mean centered. Models 2–4 were adjusted for all covariates presented by Table 1

## Discussion

The present study examined how the quality of neighborhood natural, built, and social environments influence depression across China. By exploring interactions between these environments, we have made a contribution to the literature.

Across all models, we observed that the quality of natural, built, and social environments is associated with respondents’ depressive symptoms. Regarding the natural environment, our findings indicated that more green space availability per neighborhood was positively associated with residents’ mental health. This finding is in accordance with previous studies [16, 18, 20–22, 25]. Reasonable explanations are that green spaces may increase people’s physical activity levels [38], encourage social contacts among neighbors [39], reduce stress [6, 8], and reduce environmental hazards, including air pollution and noise [6, 8].

Urbanicity was negatively associated with depressive symptoms, as found by other researchers [17, 23]. It may be that people living in a neighborhood with a higher population density are more likely to have frequent and intense contacts with neighbors and friends, both of which are known to be beneficial to mental health [17, 23]. Higher urbanicity may also be associated with better medical resources [40], so that residents living in more urban neighborhoods are likely to have better access to mental healthcare. Not in line with other studies [15, 19], that the availability of sports facilities was not associated with depressive symptoms. As confirmed by a review of previous literature [33], physical activity is a central factor in reducing the risk for depression onset or mitigating depressive symptoms. However, most respondents in our study are from rural areas, so they are more likely to have manual activity during work time and will not use sports facilities during leisure time.

We also observed that social capital was negatively associated with depression [36, 41–43], indicating that neighborhood social capital has mental health protective benefits. Only a few Chinese studies suggest that residents of neighborhoods with more social capital receive more social support from their neighbors, increasing one’s ability to cope with stress and life difficulties [44, 45]. However, neighborhood-based Gini index for income and neighborhood security had no association with mental health which is inconsistent with previous studies [27, 36]. Other Chinese studies found that income inequality increases the risk for both physical and mental disorders, as people from diverse income groups are more likely to come into conflict with each other, potentially creating a stressor that could facilitate the development of depressive symptoms [46, 47] but compared with previous studies in China, this study have much more rural respondents who may not be that sensitive to income inequality which may explain the insignificant association in terms of neighborhood security, our results is inconsistent with earlier findings from developed countries [26, 27]. This may be also because rural residents in China are not that sensitive to neighbourhood security [48].

Most importantly, the present study revealed that the social environment moderates the relationship between the built environment and depression. Regarding green space availability per neighborhood, results indicated that social capital strengthened the positive relationship between green spaces and residents’ mental health. This might be because people living in a neighborhood with pronounced social capital are more likely to be willing to share open spaces with each other or to undertake physical activities together [49], strengthening the effects of green spaces. In contrast, residents of neighborhoods with income inequality are less likely to share open spaces [26, 36]; residents may be less likely to use green spaces in such neighborhoods, reducing the positive effect of this environment. Both social capital and security also strengthened the positive association between urbanicity (i.e., population density) and mental health, while the Gini index magnitude was reduced. A possible explanation is that residents in areas with higher social capital and a safer environment are more likely to develop closer social ties, rather than having a “nodding” acquaintance with each other [24–27, 42, 49]. This could be the reason for the increased population density effect. Nevertheless, even with a higher population density, rather than having good relationships with each other, residents of neighborhoods with high income inequality are less likely to develop close social ties and may face more conflicts [26, 27]. This weakens the positive effect of population density. Finally, neighborhood-based social capital still strengthened the positive relationship between sports facilities and residents’ mental health. A reasonable explanation is that people living in neighborhoods that have higher levels of social capital and safety are more willing to share facilities and participate in sports together, instead of guarding against each other [26, 27, 43, 49]. On the contrary, residents in neighborhoods that have higher levels of income inequality may be less likely to share facilities or engage in physical activities together [26, 27]. This may weaken the positive effect of sports facilities.

The following limitations should be considered when interpreting our findings. First, restricted by data availability, our research design was cross-sectional and only reveals correlational, rather than causal, relationships. Second, neighborhoods in this study were defined as administrative units (i.e., residential committees and village committees), which may not be in line with people’s experienced neighborhood [14]. This may mean that our results could be sensitive to the underlying scale and zoning of the analyses (also known as the modifiable areal unit problem). Third, due to privacy protection regulations, we were not able to obtain the exact coordinates of respondents’ residential addresses, which would result in more accurate and objective exposure assessments. Fourth, some covariates such as physical activity may have potential mediating effect between greenspace and depression, but we only focused on the moderation effects and may ignore some potential mediation effects (for example, neighborhood social cohesion facilitation mediating the link between green space exposure and mental health) [50]. Fifth, since we mainly focused on the moderation effects, we set the relationship between environmental factors and depression to be linear, but the relationship between some environmental factors and depression may be non-linear. Last, the neighbourhood environment indicators in this study is from questionnaire. Future research should use more other source of data such as location-based big data [51–53]. Nevertheless, as one of the first Chinese studies to focus on environment–mental health associations, the present results contribute to the literature by providing novel, statistically sound, and robust empirical evidence for how broad environmental factors impact mental health outcomes.

## Conclusion

The present study confirmed that the built, natural, and social environments within a neighborhood is associated with depressive symptoms among Chines residents, while also highlighting key moderation effects. The present results suggest that the neighborhood-based social environment moderates the associations between the quality of the built/natural environment and respondents’ depressive symptoms. The present study was novel in that it also considered the interaction between different categories of neighborhood environmental factors. An interaction between the social environment strengthened the negative association between neighborhood green spaces, population density and respondents’ depressive symptoms, while a more heterogeneous income level per neighborhood mitigated the negative association. Neighborhood social capital appeared to alleviate the health benefits of green space exposure, population density, and the availability of sports facilities on respondents’ depressive symptoms, whereas income inequality within the neighborhood weakened any positive effects. We recommend that more research addressing the interactions between neighborhood environments be conducted in order to illuminate complex mental health–environment relationships.

## Data Availability

The dataset (questionnaire, individual and neighbourhood data) supporting the conclusions of this article are available in at http://css.sysu.edu.cn. To obtain more details regarding data, emails can be sent to the head of CLDS team [cssdata@mail.sysu.edu.cn].

## Abbreviations

AIC: Akaike information criterion
CES-D: Center for Epidemiologic Studies Depression Scale
CLDS: China Labour-force Dynamics Survey
VIF: Variance inflation factors
